# Correspondence between motion sensor kinematics and notational analysis of scanning in field-based football gameplay

**DOI:** 10.3389/fpsyg.2026.1785026

**Published:** 2026-07-23

**Authors:** Masahiro Kokubu, Takayuki Natsuhara, Masaaki Koido, Takumi Mieda, Takahiro Matsutake, Masao Nakayama

**Affiliations:** 1 Institute of Health and Sport Sciences, University of Tsukuba, Tsukuba, Japan; 2 Faculty of Applied Psychology, Tokyo Seitoku University, Tokyo, Japan; 3 Faculty of Education, Art and Science, Yamagata University, Yamagata, Japan; 4 Research Center for Urban Health and Sports, Osaka Metropolitan University, Osaka, Japan

**Keywords:** head scan, *in situ*, inertial measurement units, perception–action coupling, soccer, visual exploration

## Abstract

In football, players frequently engage in scanning, which consists of clearly distinguishable head-orientation changes that support visual exploration and decision-making under time pressure. While previous studies have examined scanning using video-based notational analysis or laboratory-based motion sensors, it remains unclear how scan counts based on angular velocity thresholds align with those obtained from the notational analysis during actual gameplay. This study aimed to examine the correspondence between motion sensor-based scan counts obtained using different angular velocity thresholds and scan counts obtained from video-based notational analysis during field-based football gameplay. Twelve university-level football players participated in a 6-on-6 small-sided game while wearing head-mounted inertial measurement units. Head rotation data were analyzed using four angular velocity thresholds (125, 200, 250, and 300 deg/s). Detection results were compared with the notational analysis conducted by experienced evaluators, who counted clearly distinguishable bidirectional changes in head orientation. Agreement was assessed using ANOVA, Bland–Altman plots, and concordance correlation coefficients (CCC), while detection performance was evaluated using recall, precision, and F1-score. Results showed that the 250 deg/s threshold produced scan counts that did not significantly differ from the notational analysis, yielded the smallest mean difference and narrowest limits of agreement, and achieved the highest CCC (0.932) as well as acceptable detection performance (F1-score = 0.753). In contrast, the previously used 125 deg/s threshold significantly overestimated scan counts relative to the notational analysis and demonstrated weaker agreement and lower detection accuracy. These findings indicated that, among the thresholds examined, a 250 deg/s threshold provided the closest correspondence with notational analysis when quantifying scans during field-based football gameplay. The present results suggest the importance of calibrating kinematic detection thresholds to the dynamic demands of actual sports contexts and highlight the potential of motion sensor technology for analyzing scanning *in situ*.

## 1 Introduction

In football, players must quickly perceive and process visual information to make effective decisions under time pressure. To support this, they frequently engage in scanning, characterized by head movements that expand the visual field and help players locate relevant information sources such as teammates, opponents, and the ball. While numerous studies have examined gaze strategies using eye-tracking technology (e.g., Natsuhara et al., 2020; Vaeyens et al., 2007), much of this research has been conducted in controlled laboratory settings (Roca et al., 2013), where visual search is performed under constrained and artificial conditions. Such environments may fail to reflect the complexity and dynamic nature of visual demands during actual gameplay (Aksum et al., 2021b). Although field-based investigations of visual search behavior are emerging (e.g., Aksum et al., 2020; Hüttermann et al., 2018; McGuckian et al., 2018b), studies specifically focusing on scanning in ecologically valid settings remain limited.

Recent research has used head turns to quantify scanning, a key element of visual exploration in football. Players who received individual awards at international tournaments exhibited higher scanning frequency before receiving the ball than non-award-winners (Jordet et al., 2013). Higher scanning frequency has also been associated with more frequent forward passes, suggesting a connection to offensive decision-making (Eldridge et al., 2013). Scanning also occurs more frequently before passing than during other actions such as dribbling or shooting, highlighting its role in action preparation (Jordet et al., 2020). Furthermore, analyses of UEFA youth tournaments revealed that scanning frequency was positively associated with successful passes and changes in body orientation, and that it was also related to players’ experience in national teams and opponent pressure (Pokolm et al., 2022). The direction of the final scan before ball reception was even related to which foot was used for the first touch (Pokolm et al., 2023). These findings indicate that scanning is not only frequent but also functionally relevant for action preparation in football, and responsive to contextual factors such as pressure and expertise level.

Despite valuable insights, almost all previous studies have relied on notational analysis, in which researchers manually code scans based on video footage (Jordet et al., 2013, 2020; McGuckian et al., 2018c). While this method is widely used and applicable across sports including indoor settings (Barris and Button, 2008; Doğramac et al., 2011), recent studies have also used inertial measurement units (IMUs) to detect scans from head rotation data.

Chalkley et al. (2018) developed a sensor-based algorithm for detecting head turns using an angular velocity threshold of 125 deg/s in a controlled setting. Subsequent studies have applied IMU-based approaches to football contexts (McGuckian et al., 2019), including analyses of head turns during match play (McGuckian et al., 2018b, 2020b). However, it remains unclear how scan counts obtained using laboratory-based angular velocity thresholds correspond to those obtained from video-based notational analysis under dynamic field-based gameplay conditions. This issue is important because match-play conditions involve locomotion, direction changes, and whole-body movements that may generate angular velocity unrelated to scanning, potentially affecting the correspondence between IMU-based detection and video-based notational analysis.

Studies on eye movements suggest that the definitions of fixation vary between laboratory and field studies. In laboratory settings, fixation is typically defined as a gaze that remains stable for at least 120 ms (Roca et al., 2011; Williams and Davids, 1998). However, it has been shown that players exhibit shorter fixation durations in real-world 11-vs-11 football games than reported in laboratory studies (Aksum et al., 2020). In fact, a shorter duration threshold of 60 ms has been proposed in field studies and fast-paced environments such as football (Aksum et al., 2021a). This discrepancy suggests that laboratory designs may not fully capture the dynamic environments that footballers experience in competition, and that the fixation detection threshold should be adjusted based on the context in which the study is conducted to accurately reflect real-world visual behaviors. Similarly, when analyzing head movements, applying a laboratory-based velocity threshold may lead to inaccuracies. Thus, similar contextual consideration is necessary when analyzing scanning in football.

In laboratory experiments, head movements are typically performed from a stationary position, primarily involving the neck. In contrast, real-game conditions involve head movements integrated with whole-body locomotion, such as walking, running, or changing direction, as players monitor the positions of the ball, opponents, and teammates. Thus, natural movements during locomotion may cause the head to exceed velocity thresholds established in laboratory settings. This can lead to false detections when analyzing motion sensor data in field settings. Although previous studies have employed the threshold of 125 deg/s to define head turns in controlled environments (e.g., Chalkley et al., 2018; McGuckian et al., 2019), its applicability to gameplay remains uncertain. Therefore, it is necessary to re-examine whether such thresholds are appropriate for detecting clearly distinguishable scans in real-game conditions.

The purpose of the present study was to examine the correspondence between motion sensor-based scan counts obtained using different angular velocity thresholds and scan counts obtained from video-based notational analysis during field-based football gameplay. To achieve this, we used the operational definition to align the notational analysis with the sensor-based event definition. Given that in-game head movements are likely faster and more dynamic than those in laboratory settings, we hypothesized that thresholds higher than the previously used 125 deg/s would produce sensor-based scan counts more comparable to those obtained from notational analysis under field-based gameplay conditions.

## 2 Materials and methods

### 2.1 Participants

The participants were 12 university football players (age: 20.3 ± 1.2 years; years of football experience: 14.3 ± 1.4 years; mean ± SD). The participants’ teams belonged to the top league in the district and had previously won a national championship for college students. Prior to the measurement, they were informed of the experimental protocols and provided their written informed consent to participate in the study. The protocol for the present study was approved by the Ethics Committee of Tokyo Seitoku University and Institute of Health and Sport Sciences, University of Tsukuba. All procedures were conducted in accordance with the principles of the Declaration of Helsinki.

### 2.2 Apparatus

A high-definition digital video camera (HDR-PJ800, Sony, Tokyo, Japan) was positioned at a height of 8 m above the ground and used to record the field of the modified game at a frame rate of 30 Hz. This setup allowed for an overhead view of player movements throughout the game.

**Figure 1:**
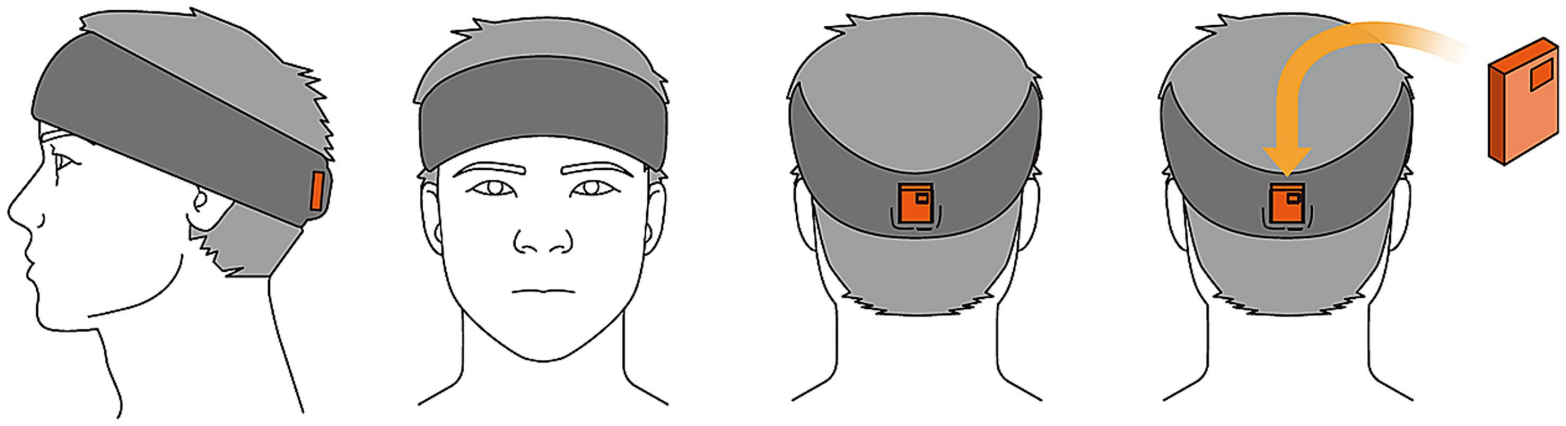
Head-mounted inertial measurement unit (IMU) used to record head kinematics during scanning. A wireless 9-axis motion sensor was attached to the participant’s head with an elastic band.

To measure scans, we utilized compact, wireless 9-axis IMUs (SS-MS-HMA16G15, Sports Sensing Co. Ltd., Fukuoka, Japan). Each IMU integrates a triaxial accelerometer (±16 G), a triaxial gyroscope (±1,500 deg/s), and a triaxial magnetometer (±10 Gauss). The sensors recorded linear acceleration, angular velocity, magnetic field strength, and quaternion orientation data at a sampling rate of 200 Hz. Each sensor measured 38 mm × 53 mm × 11 mm and weighed 24 g. A total of 12 sensors were used, with each participant wearing one sensor firmly secured to their head using an elastic headband (Figure 1).

The data from the motion sensors were synchronized and recorded using dedicated operating software installed on a laptop computer. Wireless data transmission between the sensors and the computer was facilitated by a high-power data transceiver (SS-RF24TR2, Sports Sensing Co. Ltd., Fukuoka, Japan), which supports a communication range of up to 100 m. This system enabled real-time data reception and simultaneous control of multiple sensors, ensuring precise and efficient data collection during gameplay.

### 2.3 Experimental protocol

**Figure 2:**
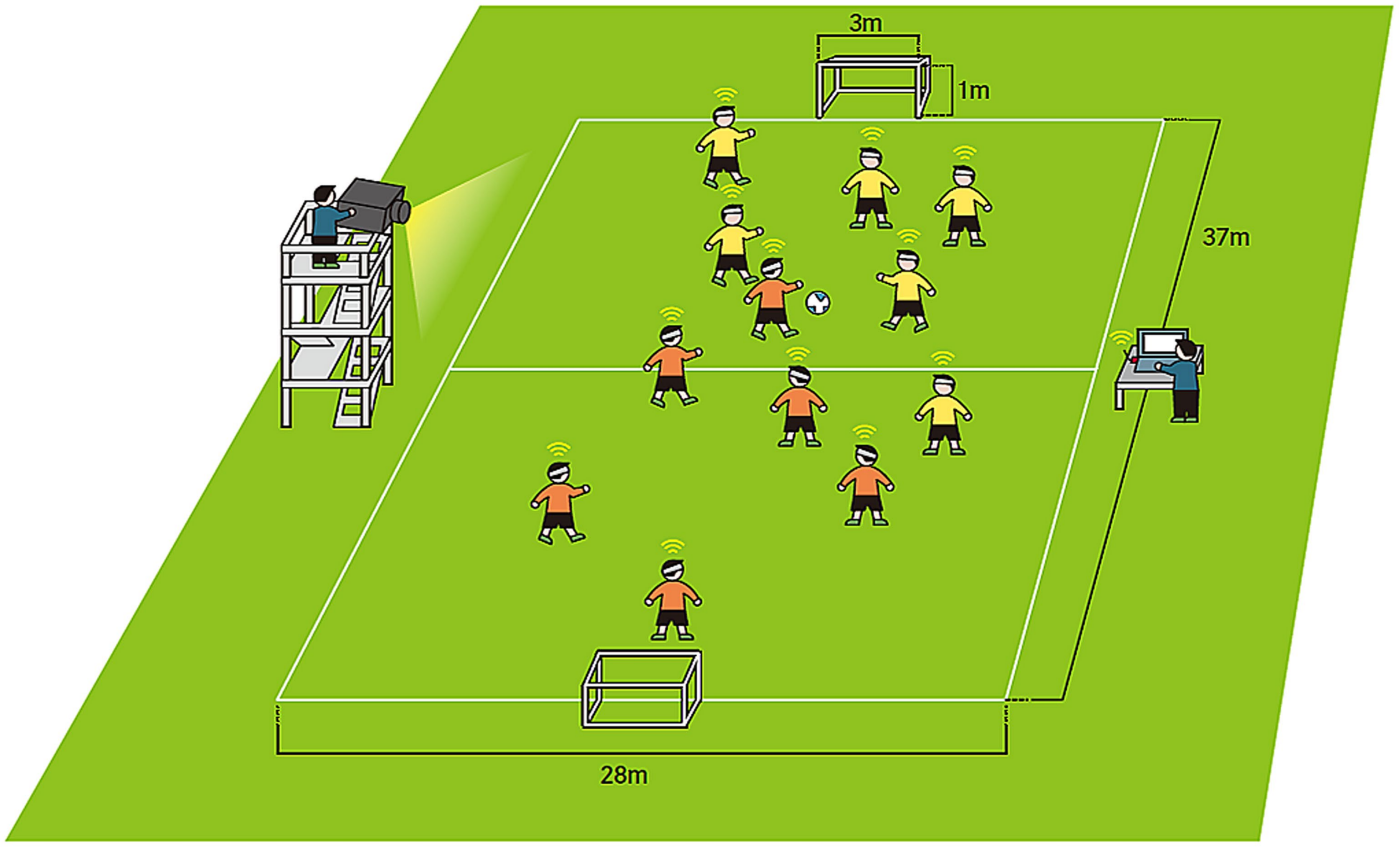
Schematic representation of the 6-on-6 small-sided and conditioned game (SSCG). The game was played on an artificial turf field (37 m × 28 m) with no goalkeepers and without an offside rule. Each team aimed to score in goals measuring 1.5 m in height and 3 m in width. During the 7-min game, participants’ motion sensor data were wirelessly transmitted to a laptop computer from 12 sensors. A video camera was positioned 8 m above the field to capture an overhead view of the entire game.

In the present study, a 6-on-6 small-sided and conditioned game (SSCG) was adopted as the experimental task as seen in earlier studies (Davids et al., 2013; Silva et al., 2014; Vilar et al., 2014). All 12 participants were outfield players, so there was no goalkeeper on the field. The aim of the game was to score as many goals as possible, and the offside rule was not applied. The conditions of the ball, playing surface, and individual playing area (IPA) were considered to ensure that the actual football environment was replicated. Specifically, the FIFA Quality Pro standard ball was used, and the experimental task was conducted on an artificial turf field measuring 37 m (length) × 28 m (width) (IPA per player: 86.3 m^2^) for 7 min (Fradua et al., 2013; Oppici et al., 2017). The size of the goal was 1.5 m (height) × 3 m (width) (Figure 2).

Before starting the game, the participants performed a warm-up for about 30 min. Just before the measurements were taken, the motion sensors were calibrated. Then, each participant wore a head-mounted IMU motion sensor secured with the elastic headband. Prior to the game, each participant was instructed to follow a predetermined sequence in which they were required to turn and orient their head to the left, to the right, upward, downward, and then return to the original position. Synchronization with the video camera was performed prior to data collection by recording the rotation of the motion sensor on video.

After completing these preparations, the participants moved to the playing field. We then confirmed that all 12 motion sensors were wirelessly transmitting data to the laptop computer and that the data were being successfully received and recorded. Once this verification was complete, the game was conducted for a duration of 7 min, during which both video and motion sensor data were recorded.

### 2.4 Data analysis

Of the 7-min recorded game, only the time periods corresponding to either attacking or defending phases were analyzed. Periods in which the ball was out of bounds or when neither team maintained possession were excluded from the dataset.

In the literature, the terminology and definitions used to describe scanning-related head motion vary across studies. Some IMU-based analyses define a “head turn” as a unidirectional rotation of the head (e.g., McGuckian et al., 2018b, 2019), whereas some video-based notational analyses define a “scan” as a bidirectional movement involving looking away from and subsequently returning toward the ball (e.g., Aksum et al., 2021a, 2021b). In the present study, we adopted a bidirectional rather than unidirectional criterion: one scan comprised a head turn away from the current orientation followed by a return or opposite-direction movement. Because the IMU-based algorithm used in the present study cannot determine ball position or gaze direction, we used the operational definition to align the notational analysis with the sensor-based event definition. Therefore, a scan was defined as a clearly distinguishable bidirectional change in head orientation, irrespective of ball position.

#### 2.4.1 Motion sensor-based analysis of scan counts

A custom software program was created using MATLAB (2024a, MathWorks, Natick, MA, USA) to quantify the number of scans performed by participants wearing the head-mounted IMU. The data processing procedure for analyzing angular velocity around the yaw axis was inspired by previous IMU-based approaches (e.g., Chalkley et al., 2018). However, the event detection criteria were modified to identify bidirectional head movements, in accordance with the definition of scanning adopted in the present study. The orientation data obtained from the IMU were represented in quaternion format and subsequently converted into Euler angles. The IMU was mounted so that the primary rotation of interest occurred around the yaw axis. To extract angular velocity along this axis, the data were smoothed using a second-order low-pass Butterworth filter with a cutoff frequency of 5 Hz. This filtering was implemented using MATLAB’s filtfilt function, which applies the filter in both forward and reverse directions to eliminate phase distortion.

**Figure 3:**
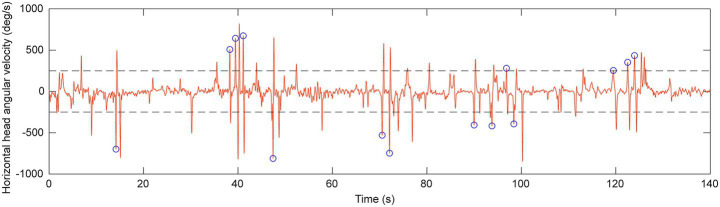
Time series data of horizontal (yaw-axis) head angular velocity from one participant during the first 140 s of the small-sided game. Positive and negative values represent rightward (clockwise) and leftward (counterclockwise) rotations, respectively. The dashed lines indicate the angular velocity threshold of ±250 deg/s used in this representative example. One scan was detected when both clockwise and counterclockwise head rotations exceeded this threshold within a 1 s window. Blue circles mark the time points at which such movements were identified. Similar detections were also performed using alternative thresholds of 125, 200, and 300 deg/s for comparative analysis.

To identify scans, a threshold-based algorithm was applied to the yaw-axis angular velocity data. The detection algorithm did not incorporate information about the position of the ball or the player’s gaze direction. Instead, scans were identified solely based on angular velocity and the bidirectional movement within a 1 s temporal window. In this study, positive values of angular velocity indicated rightward (clockwise) head rotation, whereas negative values represented leftward (counterclockwise) rotation. Four velocity thresholds were tested: 125, 200, 250, and 300 deg/s. A single scan was defined as a sequence in which the angular velocity exceeded the positive threshold in one direction and subsequently exceeded the negative threshold (or vice versa) within a 1 s window (corresponding to 200 frames). The temporal window of 1 s was selected based on prior empirical findings. Previous studies have defined long exploratory activity as a search in which the player’s face is directed away from the ball for more than 1 s before returning toward it (Jordet, 2005; Rojas Ferrer et al., 2020). However, such long-duration scans appear to be relatively rare (Jordet, 2005). Aksum et al. (2021a) also reported that 90.3% of scans during match play occurred within 0.66 s, indicating that most scanning are shorter than 1 s. Taken together, these findings support the use of a 1 s window as a threshold to capture bidirectional head movements. In addition to scan detection, peak absolute angular velocity was calculated for each identified scan to characterize the intensity of head movements. An example of this detection logic is illustrated in Figure 3 for the 250 deg/s threshold condition.

#### 2.4.2 Notational analysis of scan counts

The notational analysis of scanning was carried out independently by two evaluators, each of whom has over 10 years of experience both as competitive players and as coaches. They also hold B-level football coaching licenses certified by the national football association and have prior experience in video analysis studies. The evaluators individually counted the scans made by the 12 participants.

In previous video-based notational studies, scanning has often been defined in relation to the ball. For example, Aksum et al. (2021b) defined a scan as a self-initiated head movement in which the player’s face is temporarily directed away from the ball, presumably to look for teammates, opponents, or space relevant to subsequent action with the ball. However, the IMU-based algorithm used in the present study did not incorporate information about the position of the ball or the player’s gaze direction. Therefore, to make the notational analysis conceptually comparable with the sensor-based analysis, a scan was operationally defined as a clearly distinguishable bidirectional change in head orientation, irrespective of ball position. Accordingly, continuous movements involving a head turn in one direction followed by a return movement in the opposite direction within 1 s were counted as one scan, rather than two separate events. The evaluators were instructed to apply this operational definition consistently throughout the analysis. They were also instructed to exclude small micro-adjustments or minor fluctuations in head position, such as those associated with postural control were excluded from the analysis.

To assess reliability, inter-rater agreement for the 7-min gameplay of all participants was evaluated using the intraclass correlation coefficient (ICC). The ICC (2.1), a two-way mixed-effects model, was selected to account for the fixed evaluators and independent trial evaluations. The interpretation of ICC values followed established criteria (Koo and Li, 2016), with values below 0.50 indicating poor reliability, 0.50–0.75 moderate, 0.75–0.90 good, and above 0.90 excellent. The analysis yielded an ICC of 0.984 (95% CI: 0.935–0.996, *p* < 0.001), indicating excellent reliability. After counting the number of the scans, for events where discrepancies occurred, the two evaluators discussed each case to determine whether a scan had occurred and reached a consensus. The final count reflected only those scans on which agreement was reached.

#### 2.4.3 Comparison of scan counts between sensor-based and notational analyses

To investigate which angular velocity threshold most accurately reflects scan counts observed via notational analysis, scan counts detected by the motion sensor at each threshold was compared with the scan counts obtained through notational analysis based on video observation. Three complementary statistical approaches were employed to evaluate the level of agreement between the notational data and each sensor-based method:

1. A univariate analysis of variance (ANOVA), with condition as a fixed factor and participant as a random factor, was conducted to compare mean scan counts between notational analysis and the four sensor-based threshold conditions (125, 200, 250, and 300 deg/s). *Post hoc* comparisons were performed using Dunnett’s test, which specifically compared each sensor-based condition against the notational analysis, to identify thresholds that produced significantly different scan counts.
2. Bland–Altman analysis was conducted to assess agreement at the individual level by plotting the mean and difference of each measurement pair. For each threshold condition, the mean difference (bias) and the 95% limits of agreement (mean ± 1.96 × *SD* of the difference) were calculated. This method provides insights into the extent and direction of disagreement between methods across the full range of measurements.
3. Concordance correlation coefficients (CCCs) were computed to quantify overall agreement between sensor-based and notational measurements, incorporating both the strength of correlation and the extent of systematic bias (Lin, 1989). CCC reflects how closely the observed data conform to the line of perfect agreement (i.e., the identity line *y* = *x*). CCC values were interpreted based on established criteria (McBride, 2005), with values greater than 0.99 indicating almost perfect agreement, 0.95–0.99 substantial agreement, 0.90–0.95 moderate agreement, and less than 0.90 poor agreement.

#### 2.4.4 Performance evaluation of the detection algorithm

The performance of the sensor-based algorithm for detecting scans was evaluated by comparing its detection outcomes with those obtained through notational analysis, using a confusion matrix. Specifically, the numbers of true positives (TP), false positives (FP), and false negatives (FN) were calculated. A true positive (TP) was defined as a case in which both the sensor-based algorithm and notational analysis identified a scan. A false positive (FP) occurred when the sensor-based algorithm mistakenly detected a scan that was not identified in the notational analysis. For example, this could happen when a player looked upward at a ball passing overhead or changed direction rapidly during a transition. A false negative (FN) referred to a case in which the notational analysis identified a scan, but the algorithm failed to do so because the angular velocity did not exceed the defined threshold. To evaluate the algorithm’s detection accuracy, three performance metrics were calculated from the confusion matrix: recall, precision, and the F1 score.


Recall=TP/(TP+FN) (1)

Precision=TP/(TP+FP) (2)

F1score=2×(Recall×Precision)/(Recall+Precision) (3)

The recall rate (Equation 1) represents the proportion of cases in which both the sensor-based algorithm and the notational analysis detected scans, relative to all cases identified by the notational analysis. A higher recall rate indicates fewer missed detections by the algorithm. The precision rate (Equation 2) indicates the proportion of cases in which both methods identified scans, out of all detections made by the sensor-based algorithm. A higher precision rate reflects fewer false detections.

Based on prior literature (OʼReilly et al., 2017), recall and precision values of 0.90 or higher were considered excellent, 0.80–0.89 as good, 0.60–0.79 as moderate, and below 0.50 as poor. The F1 score (Equation 3) provides a harmonic mean of recall and precision, offering a balanced measure of algorithm performance. An F1 value of 0.70 or higher is considered as a well-performing algorithm in sports settings and acceptable in a systematic review on wearable sensors (Cust et al., 2019).

All statistical analyses were conducted using SPSS Statistics version 29 (IBM Corp., Armonk, NY, USA). The significance level was set at *α* = 0.05 and *p*-values were reported accordingly.

## 3 Results

### 3.1 Scan counts detected by motion sensor-based and notational analyses

**Figure 4:**
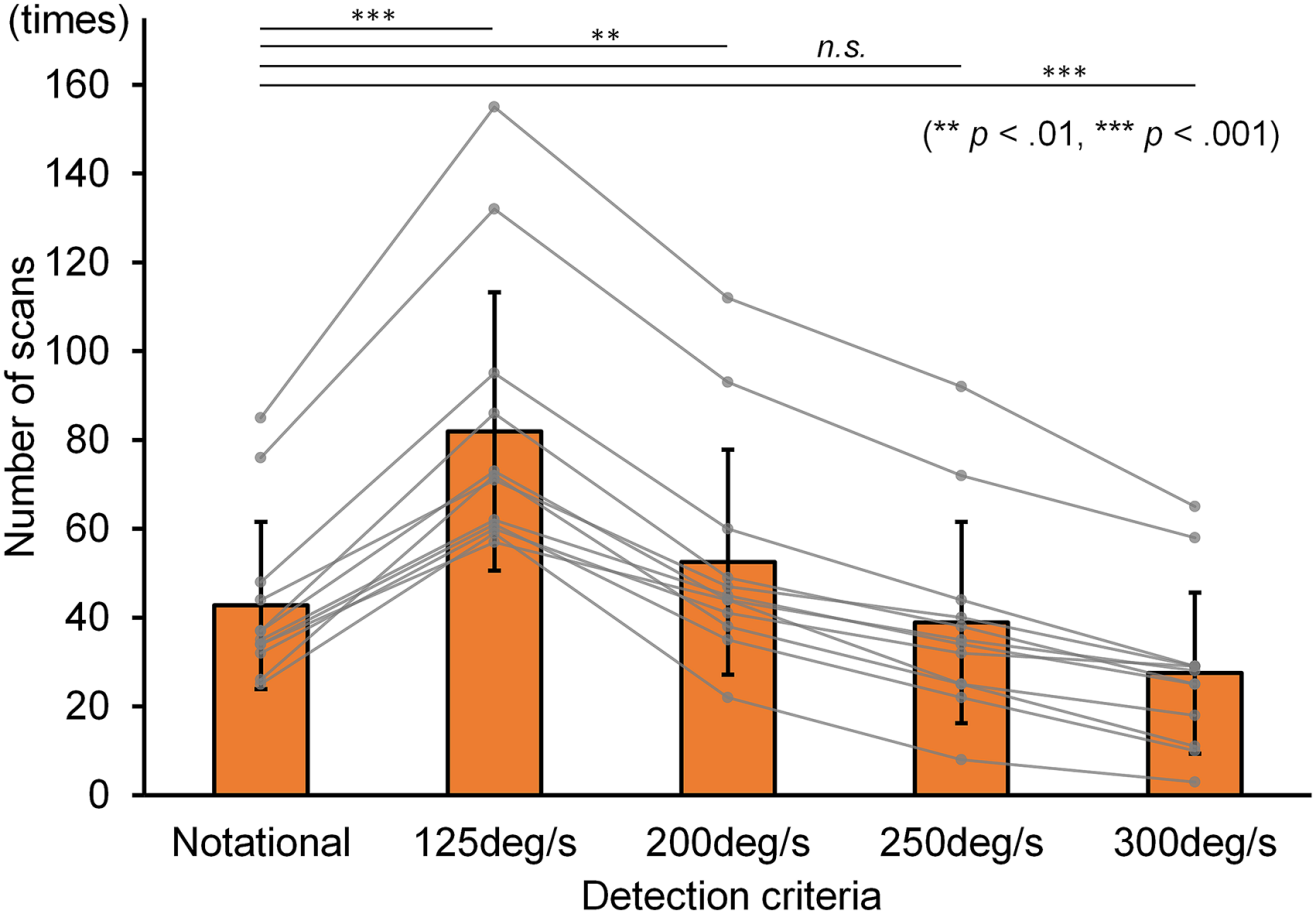
Number of scans detected using the motion sensor-based algorithm across four angular velocity thresholds (125, 200, 250, and 300 deg/s) and notational analysis. Each gray line represents an individual participant’s scan count under each detection condition. Orange bars indicate group means, and error bars represent standard deviations (*SD*). Asterisks indicate statistically significant differences compared to notational analysis (**p < 0.01, ***p < 0.001); *n.s.*, not significant.

Scan counts detected by the motion sensor-based algorithm varied according to the angular velocity threshold: 81.9 ± 31.3 at 125 deg/s, 52.5 ± 25.3 at 200 deg/s, 38.9 ± 22.7 at 250 deg/s, and 27.5 ± 18.2 at 300 deg/s. In contrast, notational analysis identified 42.8 ± 18.9 scans. Individual data for each participant are provided in Supplementary Tables S1, S2, allowing inspection of potential inter-individual differences in measurement agreement. The univariate ANOVA revealed a significant main effect of condition on the number of identified scans (*F*(4,44) = 92.994, *p* < 0.001, *η*_p_^2^ = 0.894) (Figure 4).

*Post hoc* comparisons using Dunnett’s test revealed that scan counts detected at the 250 deg/s threshold did not significantly differ from that identified by the notational analysis (*p* = 0.526). In contrast, the 125 deg/s (*p* < 0.001) and 200 deg/s (*p* = 0.009) thresholds produced significantly higher counts, whereas the 300 deg/s threshold (*p* < 0.001) resulted in a significantly lower count compared to the notational analysis.

**Figure 5:**
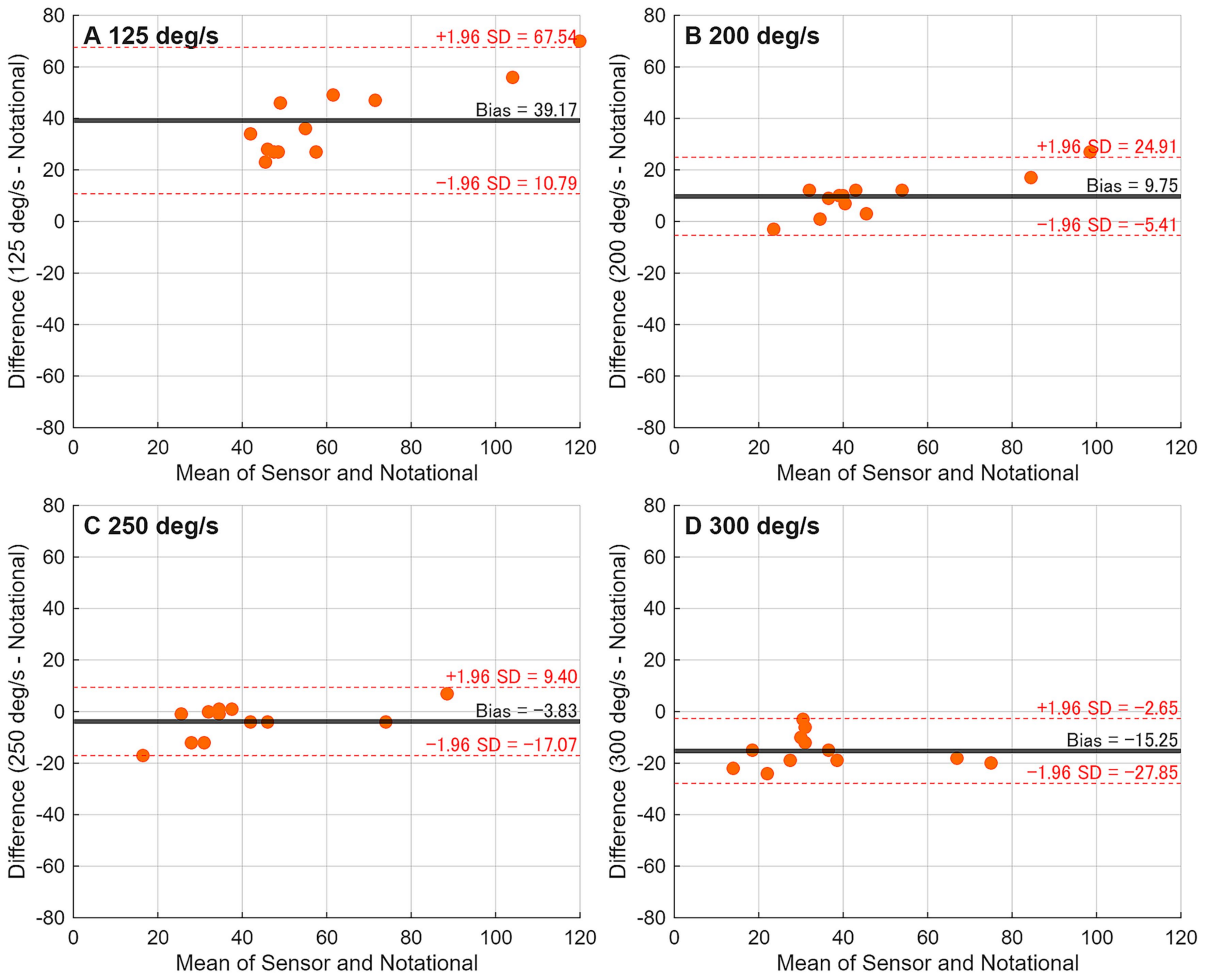
Bland–Altman plots comparing the number of scans detected by the motion sensor-based algorithm and the notational analysis across four angular velocity thresholds: **(A)** 125 deg/s, **(B)** 200 deg/s, **(C)** 250 deg/s, and **(D)** 300 deg/s. The solid horizontal line represents the mean difference (bias), while the dashed lines indicate the 95% limits of agreement (mean ± 1.96 *SD*). Each data point corresponds to an individual participant. Among the four thresholds, the 250 deg/s condition **(C)** exhibited the smallest bias and the narrowest limits of agreement, indicating the highest level of concordance with the notational analysis.

Bland–Altman analyses revealed systematic differences in scan detection across the four angular velocity thresholds. Among these, the 250 deg/s threshold (Figure 5C) demonstrated the smallest mean difference (−3.83) and the narrowest limits of agreement (−17.07 to 9.40), indicating minimal bias and low variability relative to the notational analysis. In contrast, the 125 deg/s threshold (Figure 5A) showed a substantial positive bias (mean difference = 39.17) and wide limits of agreement (10.79 to 67.54), reflecting a pronounced tendency to overestimate scans. The 200 deg/s threshold (Figure 5B) yielded a moderate positive bias (9.75) with limits of agreement from −5.41 to 24.91, suggesting a moderate degree of overestimation but less variability compared to the 125 deg/s condition. Meanwhile, the 300 deg/s threshold (Figure 5D) resulted in a negative bias (−15.25) and limits ranging from −27.85 to −2.65, indicating a systematic underestimation of scans relative to the notational analysis.

Consistent with these findings, CCCs showed that the 250 deg/s threshold exhibited the highest concordance with the notational analysis (CCC = 0.932), indicating moderate agreement. In contrast, the thresholds of 200 deg/s (CCC = 0.858), 300 deg/s (CCC = 0.702), and 125 deg/s (CCC = 0.393) all demonstrated poor agreement, with the 125 deg/s threshold showing the lowest concordance among them.

Taken together, these results indicate that the 250 deg/s threshold produced the closest estimate of scan counts relative to the notational analysis, as evidenced by both bias and agreement metrics. In addition, for the 250 deg/s threshold, the peak absolute angular velocity of detected scans across all participants was 404.2 ± 50.7 deg/s, indicating the intensity and dynamic characteristics of scans during gameplay.

### 3.2 Performance evaluation of the detection algorithm

As shown in Table 1, the performance evaluation of the detection algorithm yielded 369 TP, 98 FP, and 144 FN. Based on these values, the calculated performance metrics were as follows: recall = 0.719, precision = 0.790, and F1-score = 0.753. These results indicate that the algorithm correctly detected approximately 71.9% of scans and that 79.0% of the detected movements corresponded to actual scans.

**Table 1:** Evaluation of the performance of the algorithm for detecting scans with the sensor-based and notational analysis methods.

Metric	Value
True positives (TP)	369
False positives (FP)	98
False negatives (FN)	144
Recall	0.719
Precision	0.790
F1-score	0.753

The F1-score, which reflects the harmonic mean of recall and precision, was 0.753, indicating a reasonable balance between the two metrics. The recall value of 0.719 suggests that the algorithm detected most true scan events, with a moderate rate of missed detections. The precision of 0.790 indicates a relatively low rate of false detections. Notably, the F1-score exceeded the commonly accepted threshold of 0.70 for algorithmic validity in sports contexts (Cust et al., 2019). Taken together, these results demonstrate that the algorithm achieved a satisfactory level of performance in detecting scans during gameplay.

## 4 Discussion

The present study aimed to examine the correspondence between motion sensor-based scan counts obtained using different angular velocity thresholds and scan counts obtained from video-based notational analysis during field-based football gameplay. The results demonstrated that the 250 deg/s threshold yielded scan counts that did not significantly differ from those obtained through notational analysis. In addition, Bland–Altman analysis revealed the smallest mean difference and narrowest limits of agreement between the two methods at the 250 deg/s threshold, and CCC indicated the highest level of agreement at this threshold. Furthermore, the detection algorithm demonstrated acceptable performance at the 250 deg/s threshold, with an F1-score of 0.753. Taken together, these findings indicate that, among the thresholds examined, scan counts obtained using the 250 deg/s threshold showed the closest correspondence with those obtained from notational analysis during field-based football gameplay.

In previous laboratory-based studies (Chalkley et al., 2018; McGuckian et al., 2019), a threshold of 125 deg/s for horizontal head angular velocity has been adopted to define head turns, typically under stationary or low-range movements. However, no previous study has directly examined how scan counts obtained using this laboratory-derived threshold correspond to those obtained from notational analysis in dynamic, real-game contexts. In the current study, we hypothesized that thresholds higher than 125 deg/s would produce sensor-based scan counts that more closely corresponded to those obtained from notational analysis during actual football gameplay. Supporting this hypothesis, our findings showed that applying the 250 deg/s threshold, rather than 125 deg/s, yielded scan counts that more closely corresponded to those obtained from notational analysis. The results can be attributed to fundamental differences in movement dynamics between laboratory and field-based environments. In controlled laboratory settings, participants are often stationary and primarily rely on neck rotations to shift their gaze. In contrast, football players in game settings are frequently in motion such as walking, jogging, or sprinting while scanning their surroundings using coordinated movements involving the head, trunk, and body (Aksum et al., 2021a). Consequently, applying a low threshold such as 125 deg/s in field conditions may include head movements associated with locomotion or whole-body motion, increasing sensor-based scan counts relative to notational analysis. Consistent with this interpretation, our results showed that the 125 deg/s threshold produced significantly higher scan counts than notational analysis, highlighting the importance of calibrating detection thresholds to the movement demands of real-world gameplay.

A central issue in comparing sensor-based detection with notational analysis is the operational definition of the event being counted. Previous IMU-based studies (e.g., Chalkley et al., 2018; McGuckian et al., 2018b, 2019, 2020a, 2020b) typically defined head movements as unidirectional head turns without requiring information about the ball or gaze direction, whereas some notational studies have conceptualized scanning as bidirectional movements involving looking away from and back toward the ball (e.g., Aksum et al., 2021b). Because a head-mounted IMU alone cannot determine whether the player is looking at the ball, the present study used a shared operational definition for both analyses by counting clearly distinguishable bidirectional changes in head orientation irrespective of ball position. Therefore, the comparisons across thresholds (i.e., 125–300 deg/s) reflect differences in threshold magnitude under a consistent event definition. From this perspective, the 250 deg/s threshold should be interpreted as producing scan counts that were most comparable to those obtained from notational analysis under the operational definition used in the present field-based context, rather than as a universally optimal threshold for all possible definitions of scanning.

Importantly, the aim of the present study was not to detect all head movements, but to identify an IMU-based threshold that can detect clearly distinguishable scans under field-based football conditions. While lower thresholds may increase sensitivity, they are also more likely to capture incidental or non-exploratory head movements, such as those related to locomotion or postural adjustments. Therefore, the selection of the threshold should be understood as a balance between sensitivity and specificity in detecting behaviorally meaningful exploratory head movements. In addition, previous studies (Aksum et al., 2021a; McGuckian et al., 2018b, 2019) have often focused on head movements within short time windows (e.g., prior to ball reception), whereas the present study examined scans across the entire duration of gameplay. Under such conditions, players engage in a wider range of movements, including locomotion and transitional actions, which may increase the likelihood that lower thresholds capture incidental movements. Consistent with this, previous research (Chalkley et al., 2018) reported that IMU-based detection may yield higher counts than observer-based detection, suggesting that smaller head movements may be included when lower thresholds are applied.

Previous studies that employed a 125 deg/s threshold (Chalkley et al., 2018) did not systematically examine how scan counts obtained using this threshold correspond to those obtained from video-based notational analysis under field-based gameplay conditions. As a result, although the number of scans has been reported, the balance between over-detection and under-detection relative to notational analysis has not been fully evaluated in such dynamic contexts. The present study evaluated this relationship using recall, precision, and F1-score, which together describe the balance between missed events and additional events when comparing sensor-based detection with notational analysis. The results showed that the 250 deg/s threshold yielded an F1-score of 0.753, indicating a favorable balance between recall and precision among the thresholds examined. The interpretability of this F1-score range is supported by a previous study that used a single IMU on the lower back to distinguish fatigued from non-fatigued states, reporting F1-scores between 0.71 and 0.75 (Buckley et al., 2017). Given that both studies used a single IMU to classify specific motor states, the comparable F1-scores suggest that the algorithm used in the present study achieved a reasonable level of performance for field-based scan detection. These findings underscore the importance of evaluating detection thresholds using quantitative performance metrics when comparing sensor-based and notational analyses. By combining motion sensor data with a conceptually aligned video-based notational analysis, the present study provides a field-based framework for examining scanning in dynamic sporting environments.

Based on the algorithm using data obtained from head-mounted IMUs to detect head movements (Chalkley et al., 2018), subsequent research measured the number of scans performed by football players in the 10 s before receiving the ball during gameplay (McGuckian et al., 2018b). In addition, under controlled experimental conditions, it was demonstrated that a higher number of scans before receiving the ball and fewer scans while in possession were associated with better performance, highlighting the importance of scanning before receiving the ball (McGuckian et al., 2020a). However, because these earlier studies (McGuckian et al., 2018b, 2020a) focused only on head movements within a limited time window before ball reception, it is possible that even the lower threshold values used in laboratory experiments, such as those proposed in the previous study (Chalkley et al., 2018), were sufficient for detecting head movements in those specific contexts. In contrast, when analyzing head movements across the entire duration of a match, where gameplay is more dynamic and variable, a higher threshold may produce scan counts that more closely correspond to those obtained from notational analysis. A similar distinction has been noted in eye movement research, where a 120 ms fixation threshold is appropriate in controlled conditions, but a shorter 60 ms threshold is more suitable for full-match analyses (Aksum et al., 2021a). The findings of the present study suggest that a higher threshold may provide closer correspondence between sensor-based and notational scan counts when scanning is examined across a continuous period of field-based gameplay.

The findings of this study have several important applications and implications. The present results suggest that head-mounted IMUs may provide a useful kinematic measure of clearly distinguishable scans. Whereas video-based notational analysis has generally been used to count scans manually, wearable inertial sensors can enable the detailed and unobtrusive assessment of movement kinematics (OʼReilly et al., 2018). In football, inertial sensors have been used to quantify head movements associated with visual exploration (McGuckian et al., 2018a). Thus, in addition to providing scan counts that can be compared with notational analysis, IMUs may offer opportunities for a more detailed assessment of the kinematic characteristics of scanning, including angular velocity and movement amplitude.

While the present findings provide important insights into scanning detection during small-sided games, further consideration is needed to determine whether they can be generalized to full 11-on-11 match contexts. In full-match situations, players are exposed to greater movement variability, higher levels of kinematic noise, and more complex spatiotemporal constraints. These factors may influence both the characteristics of head movements and the correspondence between sensor-based and notational scan counts. Accordingly, the 250 deg/s threshold, which showed the closest correspondence with notational analysis among the thresholds examined in the present study, may require further examination and adjustment before being applied to more complex and dynamic match environments. Future research should investigate how contextual factors, such as playing format, positional roles, and match intensity, affect scanning and the correspondence between sensor-based and notational analyses.

Several limitations of this study should be acknowledged. First, the experimental task employed a small-sided conditioned game without a goalkeeper and without the application of the offside rule. Although this design was intended to preserve key elements of representative gameplay while allowing controlled data collection, such task constraints may have influenced players’ scanning. Experienced academy coaches have reported that visual exploration tends to occur more frequently when players perform in smaller individual playing areas (Eldridge et al., 2023). In the present 6-on-6 small-sided game conducted within a 37 × 28 m area, the restricted space may have required players to remain continuously involved in play and generally oriented toward the ball. In addition, the absence of the offside rule may have altered spatial organization and player positioning, potentially affecting the frequency or nature of exploratory movements. Future studies should examine whether similar correspondence between the two methods is observed under full-match conditions with standard rules.

Another limitation of this study is the interpretation of the F1-score. While a score of 0.7 is generally considered acceptable, a higher value would be preferable for achieving more accurate detection. To improve the accuracy of head movement detection, several strategies should be considered. One possible approach is to enhance measurement precision by optimizing sensor placement or using multiple sensors, such as attaching them to both the head and torso. Another important consideration is addressing individual differences and positional characteristics, as the speed and pattern of head movements may vary depending on the player’s position and playing style. To account for these variations, collecting a larger dataset and incorporating machine learning techniques could be beneficial. Such a data-driven approach would enable the development of personalized threshold settings tailored to each player, ultimately enhancing detection accuracy and ensuring more precise and individualized performance analysis.

## 5 Conclusion

The present study aimed to examine the correspondence between motion sensor-based scan counts obtained using different angular velocity thresholds and scan counts obtained from video-based notational analysis during field-based football gameplay. Using an aligned operational definition across the two methods, we found that the 250 deg/s threshold showed the highest level of agreement among the thresholds examined. Specifically, this threshold yielded the highest concordance correlation coefficient (0.932) and an F1-score of 0.753, indicating a favorable balance between over-detection and under-detection in comparison with notational analysis. In contrast, the previously used 125 deg/s threshold produced significantly higher scan counts and showed weaker agreement with notational analysis. These findings highlight the importance of considering the movement demands of dynamic field environments when selecting kinematic thresholds and support the potential utility of motion sensor technology for examining scanning in sport.

## Data Availability

The original contributions presented in the study are included in the article/Supplementary material, further inquiries can be directed to the corresponding author.
