# Supplementary material for: Correspondence between motion sensor kinematics and notational analysis of scanning in field-based football gameplay
Source: Front Psychol. 2026 Jul 23;17:1785026. doi: 10.3389/fpsyg.2026.1785026 (PMC13441872; doi:10.3389/fpsyg.2026.1785026)
Supplement: Supplementary file 1 [file Table_1.DOCX]

Supplementary Material

# Supplementary Tables

**Supplementary Table 1.** Inter-rater agreement for scan counts evaluated for each participant.

| Participant ID | Rater 1 | Rater 2 | Number of inconsistencies  (a) | Total events identified by either rater  (b) | Consistency rate (%)  (b-a) / (b) * 100 | Final counts after discussion between two raters |
| --- | --- | --- | --- | --- | --- | --- |
| 1 | 34 | 34 | 0 | 34 | 100 | 34 |
| 2 | 34 | 34 | 0 | 34 | 100 | 34 |
| 3 | 48 | 48 | 6 | 51 | 88.2 | 48 |
| 4 | 46 | 41 | 7 | 47 | 85.1 | 44 |
| 5 | 25 | 26 | 1 | 26 | 96.2 | 26 |
| 6 | 39 | 36 | 5 | 40 | 87.5 | 37 |
| 7 | 35 | 35 | 0 | 35 | 100 | 35 |
| 8 | 32 | 32 | 0 | 32 | 100 | 32 |
| 9 | 37 | 34 | 3 | 37 | 91.9 | 37 |
| 10 | 85 | 83 | 4 | 86 | 95.3 | 85 |
| 11 | 79 | 70 | 11 | 80 | 86.3 | 76 |
| 12 | 25 | 25 | 0 | 25 | 100 | 25 |
| Mean | 43.3 | 41.5 | 3.1 | 43.9 | 94.2 | 42.8 |
| SD | 19.4 | 17.6 | 3.6 | 19.8 | 6.1 | 18.9 |

**Supplementary Table 2.** Scan counts for each participant using motion sensor-based analysis across four angular velocity thresholds, compared with notational analysis.

| Participant ID | Team ID | Position | Notational | 125 deg/s | 200 deg/s | 250 deg/s | 300 deg/s |
| --- | --- | --- | --- | --- | --- | --- | --- |
| 1 | 1 | FW | 34 | 57 | 44 | 35 | 28 |
| 2 | 1 | MF | 34 | 61 | 35 | 22 | 10 |
| 3 | 1 | MF | 48 | 95 | 60 | 44 | 29 |
| 4 | 1 | MF | 44 | 71 | 47 | 40 | 29 |
| 5 | 1 | DF | 26 | 72 | 38 | 25 | 11 |
| 6 | 1 | DF | 37 | 73 | 44 | 25 | 18 |
| 7 | 2 | FW | 35 | 62 | 45 | 34 | 25 |
| 8 | 2 | MF | 32 | 60 | 41 | 32 | 29 |
| 9 | 2 | MF | 37 | 86 | 49 | 38 | 25 |
| 10 | 2 | MF | 85 | 155 | 112 | 92 | 65 |
| 11 | 2 | DF | 76 | 132 | 93 | 72 | 58 |
| 12 | 2 | DF | 25 | 59 | 22 | 8 | 3 |
| Total |  |  | 513 | 983 | 630 | 467 | 330 |
| Mean |  |  | 42.8 | 81.9 | 52.5 | 38.9 | 27.5 |
| SD |  |  | 18.9 | 31.3 | 25.3 | 22.7 | 18.2 |

Note: FW = forward; MF = midfielder; DF = defender.
